# The effects of insulin-regulated aminopeptidase inhibition with HA08 on recognition memory acquisition and spatial working memory under reversed circadian conditions in male rats

**DOI:** 10.1016/j.ibneur.2025.10.022

**Published:** 2025-11-02

**Authors:** Frida Stam, Sara Bjurling, Sofia Zelleroth, Sally Badrd'din, Johan Gising, Mats Larhed, Mathias Hallberg, Alfhild Grönbladh

**Affiliations:** a The Beijer Laboratory, Department of Pharmaceutical Biosciences, Neuropharmacology and Addiction Research, Uppsala University, Uppsala, Sweden; b The Beijer Laboratory, Science for Life Laboratory, Department of Medicinal Chemistry, Uppsala University, Uppsala, Sweden

**Keywords:** Cognition, Behaviour, Insulin-regulated aminopeptidase (IRAP), Intracerebroventricular injection, Memory, Novel object recognition (NOR), Y-maze

## Abstract

In 1995, a new M1 aminopeptidase member was discovered in fat and muscle cells, the insulin regulated aminopeptidase (IRAP; oxytocinase; placental leucine aminopeptidase). IRAP can be found in glucose transporter type 4 (GLUT4) vesicles and is believed to regulate cellular glucose uptake by mediating trafficking of GLUT4 to the plasma membrane. It is expressed in various tissues, including areas of the brain associated with cognition, and it cleaves multiple endogenous substrates like oxytocin, vasopressin, somatostatin and cholecystokinin-8. In the beginning of the century, IRAP was identified as the receptor of hexapeptide angiotensin IV (AngIV). AngIV and similar ligands binds to the active site of the peptidase, causing inhibition of its enzymatic activity, which is suggested to increase neuropeptide levels and modulate cellular glucose uptake. This mechanism is suggested to improve cognitive functions, and in 1988 the first evidence of Ang IV’s memory enhancing effects was reported. Since then, several animal behaviour models have demonstrated the positive effects of AngIV on memory. One of the most potent synthetic IRAP inhibitors known today is the macrocyclic compound HA08, that we have previously demonstrated to increase dendritic spine density and restore cell viability. To further evaluate the potential of IRAP inhibitor HA08 as a cognitive enhancer, we have examined this macrocycle compound *in vivo* using male Sprague Dawley rats. The effect of a single acute intracerebroventricular injection with HA08 on recognition memory acquisition and spatial working memory was evaluated by conducting a novel object recognition test and a Y-maze test. The overall results of this study suggest that an acute single dose of HA08 does not influence memory acquisition in the novel object recognition test, nor spatial memory using a Y-maze, in male rats with intact cognitive functions tested under a reversed light-dark cycle.

## Highlights

• Insulin-regulated aminopeptidase (IRAP) is linked to cognition and neuroprotection.
• HA08 is a potent macrocylic IRAP inhibitor with neuroprotective effects *in vitro*
• Acute i.c.v. HA08 was evaluated for effects on recognition and spatial memory.
• Novel object recognition and Y-maze used to assess memory performance in rats.
• HA08 did not alter memory performance under the conditions of this study.

## Introduction

**Fig. 1:**
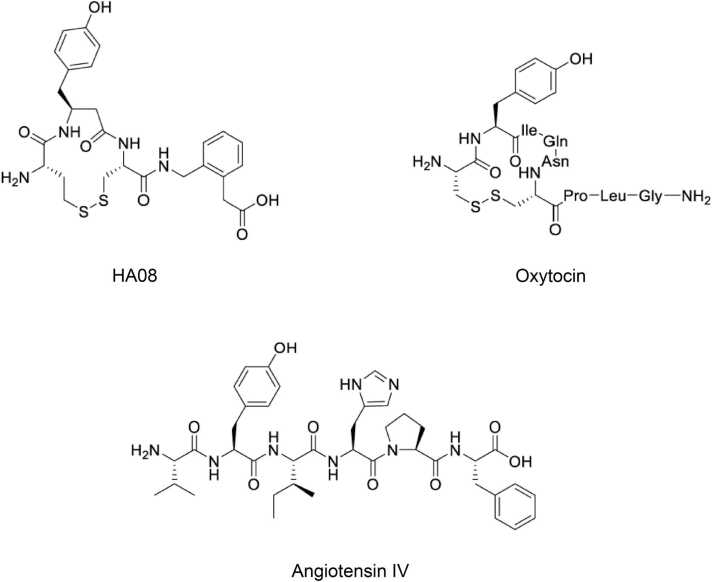
Chemical structures of the synthetic IRAP inhibitor HA08 (K_i_ = 3.3 nM), oxytocin, and angiotensin IV (K_i_ = 62.4 nM) (Andersson et al., 2010).

In 1995, a new M1 aminopeptidase member, insulin regulated aminopeptidase (IRAP; oxytocinase; placental leucine aminopeptidase) was discovered in fat and muscle cells by Keller et al. (1995). It is expressed in various tissues, including areas of the brain associated with cognition (Chai et al., 2000, Fernando et al., 2005, Matsumoto et al., 2001). IRAP can be found in glucose transporter type 4 (GLUT4) vesicles and is believed to regulate cellular glucose uptake by mediating trafficking of GLUT4 to the plasma membrane (Bryant et al., 2002, Keller, 2003, Seyer et al., 2020, Waters et al., 1997), and it cleaves multiple endogenous substrates like oxytocin, vasopressin, somatostatin and cholecystokinin-8 (Albiston et al., 2003, Chai et al., 2004, Herbst et al., 1997, Matsumoto et al., 2001, Rogi et al., 1996). This diverse peptidase is also involved in the immune system, specifically in major histocompatibility complex (MHC) class I antigen presentation and T cell activation (Evnouchidou et al., 2020, Saveanu et al., 2009). Although IRAP is associated with various physiological functions, the exact role of this peptidase remains unknown. In the beginning of the century, IRAP was identified as the receptor of hexapeptide angiotensin IV (AngIV) (Albiston et al., 2001, Chai et al., 2004). AngIV and similar ligands binds to the active site of the peptidase, causing inhibition of its enzymatic activity which is suggested to increase neuropeptide levels and modulate cellular glucose uptake (Albiston et al., 2003, Bryant et al., 2002, Chai et al., 2004, Seyer et al., 2020, Waters et al., 1997). This mechanism is suggested to improve cognitive functions, and in 1988 Braszko et al. reported the first evidence of AngIV having memory enhancing effects (Braszko et al., 1988). Since then, several animal behaviour models have demonstrated the positive effects of AngIV on memory (Albiston et al., 2011, De Bundel et al., 2009, Gard, 2008, Kilic et al., 2021, Paris et al., 2013, Royea et al., 2020, Wright et al., 1999). This has inspired the search and development of new IRAP-inhibiting ligands, in a pursuit for cognitive enhancers that are more suitable as pharmaceuticals compared to the endogenous hexapeptide AngIV (Albiston et al., 2008, Andersson and Hallberg, 2012, Axén et al., 2006, Gising et al., 2024, Hallberg, 2009, Hallberg and Larhed, 2020). One of the most potent IRAP inhibitors known today is the macrocyclic compound HA08 developed and synthesized by Andersson et al. in 2010 (compound 8; (Andersson et al., 2010)). The structure of this macrocycle combine elements from Ang IV and the physiological substrates oxytocin and vasopressin (Fig. 1), and it binds competitively to the catalytic site of IRAP (Mpakali et al., 2020). HA08 has a high binding affinity to the IRAP receptor and it has previously been demonstrated by our research group that this inhibitor increases dendritic spine density (Diwakarla et al., 2016a) and can restore cellular viability *in vitro* (Stam et al., 2022), suggesting a potential modulation of cognitive functions.

As previously mentioned, the hexapeptide Ang IV has been demonstrated to improve memory functions in several behaviour paradigms, including spontaneous alternation tasks and object recognition tasks in rodents (Braszko, 2004, De Bundel et al., 2009, Paris et al., 2013). Improved spontaneous alternation and object recognition has also been reported after treatment with synthetic IRAP inhibitor HFI-419 (Albiston et al., 2008, Seyer et al., 2020). The novel object recognition test is commonly used to study recognition memory, and is based on the rodents innate curiosity to explore new things (Antunes and Biala, 2012, Cohen and Stackman, 2015). Spontaneous alternation tests are based on the same innate exploratory behaviour, and are used to study spatial working memory with the use of various maze arenas, for example the Y-maze (Kraeuter et al., 2019, Lalonde, 2002).

To further evaluate the potential of IRAP inhibitor HA08 as a cognitive enhancer, we have examined this macrocycle compound *in vivo* using male Sprague Dawley rats. The effect of acute treatment with HA08 on recognition- and spatial working memory was evaluated by conducting a novel object recognition test and a Y-maze test.

## Methods

### Animals

**Fig. 2:**
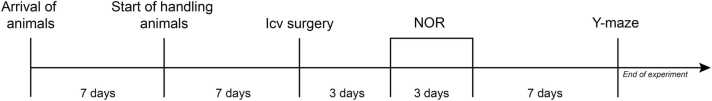
Schematic time-line of study design. The animals arrived at the animal facility at 7 weeks of age. After one week, handling of the animals started and after another 7 days intracerebroventricular (icv) surgery was performed. The animals rested for 3 days before the first phase of the novel object recognition (NOR) test was started. One week after the NOR test was finished, a subgroup of rats performed an additional behaviour test, the Y-maze.

All animal experiments in this study were executed in accordance with Swedish rules and guidelines for animal experiments (Animal Welfare Act SFS: 2018:1192) and the European Union directive on the Protection of Animals Used for Scientific Purposes (Directive 2010/63/EU). Male Sprague Dawley rats (n = 49; Envigo, Netherlands) were used to conduct the behaviour studies and the experiment protocol was approved by Uppsala local animal ethics committee (permit number 5.8.18–15660/2019). The animals arrived at the animal house 7 weeks old and was housed with one litter mate in transparent type VI cages (59 × 38 × 20 cm), bottom area of 1820 cm^2^, with raised cage lid, wood chip bedding (Tapvei Estonia OÜ, Estonia), a wooden house, and with standard rat pelleted food (ssniff Spezialdiäten GmbH, Germany) and water provided ad libitum. The rats were acclimatized for two weeks and kept on a reversed circadian rhythm (lights off at 07:00, lights on at 19:00). The temperature was controlled to 20 ± 1 °C, with 40 ± 10 % humidity. Each animal was handled and weighed continuously throughout the trial. At the age of 9 weeks, intracerebroventricular surgery was performed to attach a cannula guide implant to enable intracerebroventricular injection of IRAP inhibitor HA08. Three days after surgery the behaviour testing was initiated during which the treatment was given. See Fig. 2 for a schematic time-line of the study design.

### IRAP inhibitor HA08

The macrocyclic IRAP inhibitor HA08 (Andersson et al., 2010) was used for treatment of the Sprague Dawley rats. The substance was dissolved in 100 % dimethyl sulfoxide (DMSO) to a concentration of 1 × 10^−2^ M and stored in - 20°C. Prior to treatment start, the stock solution was thawed and further diluted in saline into working concentration 1 × 10^−8^ M (0.0001 % DMSO). The control animals were treated with saline.

### Intracerebroventricular surgery

To enable direct administration of HA08 or control substance to the brain, the animals were provided with a cannula guide implant in the right cerebral ventricle. The implant was provided to each animal during intracranial surgery performed three days before behavioural testing. There was also a group of animals included in the novel object recognition study that did not undergo any surgery (untreated animals), to evaluate potential behaviour effects of the surgery. The rats were sedated with 5 % isoflurane in an isolated box before being placed on a heating pad and mounted in a stereotaxic instrument with a mouth piece providing isoflurane (1.5–2 %) continuously during the entire surgery. Local analgesia (bupivacaine 2.5 mg/ml) was distributed subcutaneously around the head before the skull was exposed. The cannula guide (C315G; Bilaney consultants GmbH, Düsseldorf, Germany) was placed in the right cerebral ventricle by drilling a hole at flat skull coordinates 0.8 mm posterior to bregma, and 1.5 mm lateral to the midline. The guide was placed 3.5 mm ventral to the surface of the dura mater and fixed with stainless-steel screws (AgnThos AB, Lidingö, Sweden) and dental cement (Cenger Scandinavia AB, Väröbacka, Sweden). A capped dummy cannula (C315DC; Bilaney consultants GmbH), reaching an additional 1.0 mm further down in the ventricle, was placed in the guide until injection. The wound was closed around the implant with sutures, and systemic analgesia (carprofen 5 mg/kg) was given subcutaneously before removing the rat from the stereotaxic instrument and placing it in a recovery box with a heating pad. The animals were single housed following the surgery, and behavioural testing started three days after the surgery.

### Intracerebroventricular injection with HA08

During behaviour testing, the rats were injected with an acute dose of 1 × 10^−8^ M HA08, or control substance saline, directly in the right cerebral ventricle. The dummy cannula was removed from the guide opening, and a cannula for injection (C315I; Bilaney consultants GmbH) was carefully placed in the guide instead. The cannula was connected to one end of a plastic injection tube, and the other end of the tube was connected to a Hamilton™ syringe. The animal was held still while the cannula was placed in the guide and during injection to make sure that all the substance was successfully transferred into the implant. The animal was injected with a volume of approximately 5 µl in total;1 µl saline, followed by 3 µl substance (HA08 or saline), followed by an additional 1 µl saline. To ensure correct placement of the guide for injection, a control dye (methylene blue) was injected post euthanization and brains were collected for visual inspection of staining in the ventricles.

### Novel object recognition test

**Fig. 3:**
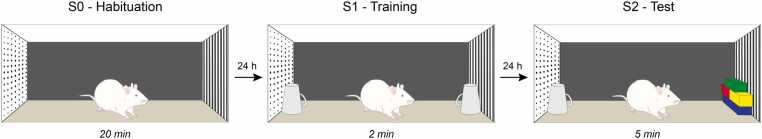
Study design of the novel object recognition test. Male Sprague Dawley rats were used to evaluate the acute treatment effect of IRAP inhibitor HA08 using the novel object recognition (NOR) test. Intracerebroventricular surgery was performed to enable direct injection in the ventricle. The NOR test was divided into three phases performed over three days. The first phase (S0) was the habituation phase with a duration of 20 min, followed by the training phase (S1) where the animal spent 2 min freely exploring two identical objects placed in the arena. The last phase (S2), performed on the third day, was the test phase where the animal spent 5 min freely exploring one familiar object from the S1 phase and one novel object. The discrimination ratio of the test phase was calculated by dividing the time spent investigating the novel object with the total time spent investigating both objects.

The novel object recognition test was used to determine the effect of HA08 on memory performance. This test is commonly used to study recognition memory and is based on the rats innate curiosity to explore new things (Antunes and Biala, 2012, Cohen and Stackman, 2015). The arena used for the test was a square box with dimensions 80 cm long, 31 cm wide, 53 cm tall, and made of black plastic. On the short ends of the arena, there were cues in the form of dots and stripes. The bottom was covered with shavings from each home cage of the animals that were tested. The light intensity was 9 lux in the centre of the arena. The NOR test was designed based on a previously described protocol (Feltmann et al., 2015), and divided in three phases, phase S0 - the habituation phase; phase S1 - the training phase and phase S2 - the test phase. The phases were executed over three consecutive days. In the first phase, S0, the rat spent 20 min freely investigating the empty arena. Five minutes before starting the second phase, S1, on the second day, 3 µl HA08 was injected directly in the ventricle of the rat through the cannula implant. The control group was injected with saline. In phase S1 two identical objects were placed at each end of the arena, and the rat spent 2 min freely investigating the arena. In the last phase, S2, performed on day three, one of the familiar objects from S1 was replaced with a novel object, and the rat spent 5 min freely investigating the arena. See Fig. 3 for study design of the NOR test. After the performed test, the discrimination ratio of the time spent investigating the new object and the total time investigating both objects was calculated. Object investigation was defined as the animal facing the object and actively exploring it (the nose minimum one cm from the object). The objects used in the test was either a block of lego or a cone-shaped cup with an ear (lego or cup as novel object was equally distributed between the treatments groups), and the scoring of object investigation was performed by a treatment-blinded investigator. The animals performed the test during their dark cycle (active cycle). All of the negative control group animals were discontinued from the study after the NOR test.

### Y-maze test

**Fig. 4:**
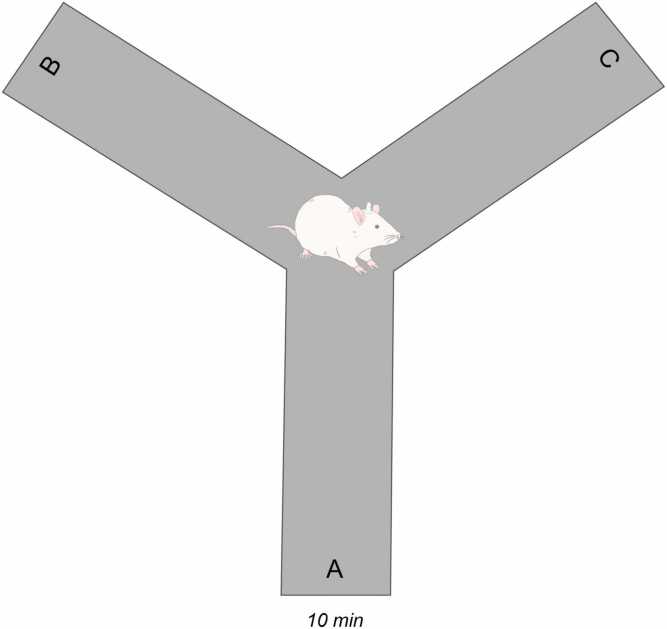
Study design of the Y-maze. Male Sprague Dawley rats were used to evaluate the acute treatment effect of IRAP inhibitor HA08 on spontaneous alternation using the y-maze. Intracerebroventricular surgery was performed to enable direct injection in the ventricle. The Y-maze was performed as one single session where the animal spent 10 min freely investigating the maze. The arm entry sequence was recorded to evaluate how many of the arm alternations that were correct to calculate the spontaneous alternation behaviour score.

After performing in the novel object recognition test, a subgroup of the rats continued with an additional behaviour test, the Y-maze, one week later. The rats were treated with 10^−8^ M of IRAP inhibitor HA08 diluted in saline and used for the Y-maze test to further evaluate the effects of HA08 on spatial working memory performance. The arena was a three-armed maze with 120° angle (arm A, B and C, see Fig. 4). Each arm was 50 cm long, 10 cm wide, and 20 cm tall. The light intensity in the centre of the arena was 9 lux. The test was performed as one single session of 10 min freely exploring the arena. Five minutes before starting the test, HA08 was injected directly in the ventricle of the rat through the cannula implant. The control group was injected with saline. The arm entry sequence was recorded to evaluate how many of the arm alternations that were correct. A correct arm alternation means that the rat visits a new arm and do not return to the arm it last visited. Example of a correct arm alternation is A-B-C or B-A-C and an incorrect alternation is A-B-A or B-A-B. The spontaneous alternation behaviour score (SAT) was calculated by dividing the number of correct 3-choice alternations with the total number of chances of 3-choice alternations (Momeni et al., 2015). The animals performed the test during their dark cycle (active cycle).

### Statistical analysis

GraphPad Prism (version 10.2.3) was used to perform all statistical analysis. The data collected from the behavior studies comparing 2 groups was analysed using an unpaired *t*-test. Data comparing multiple groups (>2) was analysed using one-way ANOVA, and if there was a significant overall effect this was followed by Dunnett’s post hoc test with comparison to the negative control group. All data is expressed as means ± standard deviation (SD) and statistical significance was defined as p-value < 0.05.

## Results

### Novel object recognition test

**Fig. 5:**
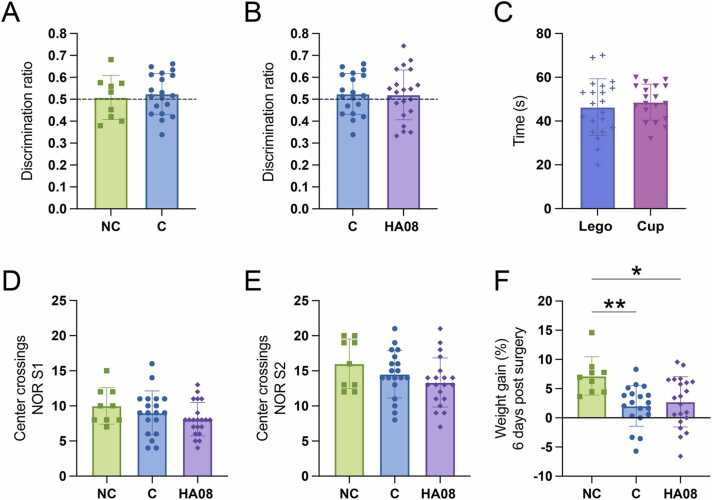
Results of the novel object recognition test. Male Sprague Dawley rats were evaluated for recognition memory in the novel object recognition (NOR) test after acute treatment with IRAP inhibitor HA08 (1 ×10^−8^ M). The control group (C) was treated with saline. A negative control group (NC) that did not undergo any surgery, and received no treatment, was also included in the study. Discrimination ratio 0.5 is marked with a dotted line in graph A and B. **A**) There was no difference in the discrimination ratio of phase S2 between the negative control group (NC; n = 9) and the control treated group (C; n = 19). **B**) There was no difference in the discrimination ratio in phase S2 between the control group (n = 19) and the animals treated with HA08 (n = 21). **C**) There was no difference in the total time spent investigating the objects in phase S1 between the two objects used in the test, lego (n = 21) and cup (n = 19). **D)** The number of crossings of the centre of the NOR arena was recorded and compared between the treatment groups. There was no overall treatment effect on the number of centre crossings in phase S1 (NC; n = 9, C; n = 19, HA08; n = 21). **E)** The number of crossings of the centre of the NOR arena was recorded and compared between the treatment groups. There was no overall treatment effect on the number of centre crossings in phase S2 (NC; n = 9, C; n = 19, HA08; n = 21). **F**) All animals were weighed continuously throughout the study and the weight gain from the day of surgery to the day of the NOR S2 test phase was calculated in percentage. There was a significant difference in total weight gain between the animals that underwent surgery (HA08;n = 21, control group; n = 19) compared to the animals that did not have surgery (NC group; n = 9). All data are presented as means ± SD, * p-value < 0.05, **p-value < 0.01.

The results from the S2 test phase in the novel object recognition test was converted to a discrimination ratio by dividing the time spent investigating the novel object with the total time spent investigating both objects. The results showed that there was no significant difference in discrimination ratio between the control animals and the HA08 treated animals (p-value 0.9004), see Fig. 5B. The mean discrimination ratio for the HA08 group was 0.520, and the mean for the control group was 0.524. Both groups had a ratio very close to 0.5, indicating that they spent equal amount of time investigating both the familiar object and the novel object. The effect of the intracerebroventricular surgery and placement of the cannula on memory performance in the NOR test was also considered, and a negative control group was therefore included. These animals did not undergo any surgery and received no injection, and there was no difference in S2 discrimination ratio for this group when compared to the control treated group (p-value 0.9992), see Fig. 5A. Furthermore, to exclude that there was any preference for any of the used objects (lego or cup), the time spent investigating the objects during the training phase, S1, was determined for each animal and compared as groups ‘Lego’ and ‘Cup’ (Fig. 5C). There was no difference in investigation time between the groups (p-value 0.51976), indicating that the animals did not prefer the lego object over the cup object or vice versa. To assess the activity of the different treatment groups, the number of times the rat crossed the center of the arena was recorded for phase S1 and S2. There was no significant treatment effect on the number of center crossings between the three groups in any of the phases (Figs. 5D and 5E). The animals were also weighed continuously throughout the study, and the weight gain from the day of surgery to the last day of NOR (6 days post-surgery) was calculated in percentage. There was an overall treatment effect on the weight gain (ANOVA p-value 0.0055), and further post-hoc analysis revealed that both the control animals and the HA08 treated animals had a significant decrease in weight gain compared to the negative control animals (p-values 0.0037 and 0.0115, respectively), indicating that the surgery had an impact on the animals weight gain during the first 6 days after surgery (Fig. 5F).

### Y-maze test

**Fig. 6:**
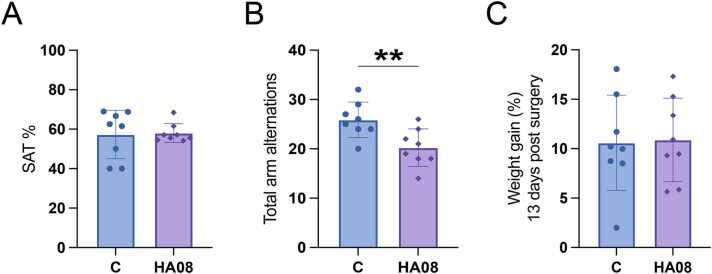
Results of the Y-maze test. Male Sprague Dawley rats were evaluated for spatial working memory in the Y-maze test after acute treatment with 1 × 10^−8^ M IRAP inhibitor HA08 (n = 8). The control group (C; n = 8) was treated with saline. **A)** The spontaneous alternation behaviour score (SAT) was calculated by dividing the number of correct 3-choice alternations with the total number of chances of 3-choice alternations. There was no effect on the SAT score after treatment with 10^−8^ M HA08 compared to the control treated group. **B)** The total number of arm alternations was recorded and the HA08 group had a significantly lower number of arm alternations compared to the control group. **C)** All animals were weighed continuously throughout the study and the weight gain from the day of surgery to the day of the Y-maze was calculated in percentage. There was no significant difference in total weight gain between the HA08 treated group and control animals. All data are presented as means ± SD, **p-value < 0.01.

After performing in the novel object recognition test, a randomised subgroup of the animals continued the behaviour study by performing in a Y-maze test one week later. Only the animals from the control group (saline injected) and the HA08 treated group was continued to the Y-maze. Five minutes prior to performing in the maze, these animals were given a second intracerebroventricular injection with either HA08 or control substance saline. The spontaneous alternation behaviour score (SAT) was calculated by dividing the number of correct 3-choice alternations with the total number of chances of 3-choice alternations. There was no difference in SAT percentage between the control treated animals and the HA08 treated animals (p-value 0.8816), see Fig. 6A. The mean SAT for HA08 treated animals was 58 %, and the mean for control treated animals was 57 %. To assess the activity of the two different treatment groups, the number of total arm alternations was recorded which revealed a significant difference between the groups (p-value 0.0089). The rats that were treated with IRAP inhibitor HA08 made fewer arm alternations during the test compared to the saline treated rats (Fig. 6B). The animals were also weighed continuously throughout the study, and the weight gain from the day of surgery to the day of the Y-maze test (13 days post-surgery) was calculated in percentage. There was no treatment effect on the total weight gain between the two groups (Fig. 6C).

## Discussion

The overall results of this study demonstrate that an acute single dose of HA08 does not impact memory acquisition in the novel object recognition test, or performance in a spatial memory test using Y-maze, in male Sprague Dawley rats with intact cognitive functions tested during a reversed light-dark cycle. The behaviour tests used in this study are based on the rodents innate curiosity to explore new things and surroundings, and they are commonly used to evaluate effects on recognition and spatial working memory (Antunes and Biala, 2012, Cohen and Stackman, 2015, Kraeuter et al., 2019). The NOR test can be designed in different ways, here we used a rectangle shaped arena where the objects were placed in each end of the arena. The discrimination ratio was approximately 0.5 for HA08 treated animals and the control animals, which indicates that none of the groups remembered the familiar object in the S2 test phase. The results also showed that there was no preference for any of the objects used in the NOR test, lego and cup, and that each rat was curious to explore the objects. In a previous study, where male Sprague Dawley rats were treated with an acute single intracerebroventricular (icv) dose of IRAP inhibitor HFI-419, improved recognition memory was demonstrated using a slightly different setup of the novel object test, mainly regarding the time frame of each phase (Albiston et al., 2008). Additionally, the same study also demonstrated improved spontaneous alternation in a plus maze task following HFI-419 administration, which was further confirmed by Seyer et al. in 2020 (Albiston et al., 2008, Seyer et al., 2020).

Interestingly, a HA08-induced decrease in the rats’ locomotor activity (total arm alternations) was observed during performance in the Y-maze. This was following a second HA08 injection, with 8 days in between the icv administrations. The impact of these results is difficult to interpret as the cognitive performance seem to remain equal between HA08 and saline treated animals. However, a decreased locomotor activity could indicate alterations in other behavioural domains, such as increased anxiety-like behavior, sedation, or dysfunction in motor-related circuits. The IRAP inhibitor treatment demonstrated no change in recognition memory or spontaneous alternation in our study, which could possibly be explained by several factors, such as dose, administration interval, and behavioural test design. Considering that a bell-shaped dose response trend has been seen for HA08 in previous *in vitro* studies (Diwakarla et al., 2016a, Stam et al., 2022), the 10 nM dose used could be insufficient, or a single dose is not adequate enough to induce measurable cognitive effects under these circumstances. Several *in vitro* studies with synthetic IRAP inhibitors, including HA08, have demonstrated treatment effect on spine density in intact primary cell cultures after repeated treatment during several days (Diwakarla et al., 2016a, Diwakarla et al., 2016b, Seyer et al., 2020, Stam et al., 2024). The dose used in our study was determined based on previous *in vitro* findings, as well as previous studies using icv injections for IRAP inhibitor treatment. Interestingly, Albiston et al. has demonstrated a probable bell-shape dose response for HFI-419 *in vivo*, where a higher dose (1 nM) of the inhibitor had no effect on spontaneous alternation and the lower dose (0.1 nM) improved this ability (Albiston et al., 2008). Furthermore, the variation in behaviour test design can significantly impact the outcome of the memory performance measurements. For the NOR test, they used a longer training phase (S1) and a shorter test phase (S2) compared to our setup, which could be beneficial for recognition establishment of the objects. In our maze study, the animals freely explored a maze consisting of three arms for 10 min, whereas the above-mentioned studies with HFI-419 used a four-arm plus maze and the animals were recorded exploring for 20 min. A longer exploration phase could potentially be more beneficial for evaluating rodent exploratory behaviour based on their curiosity, as the animals tend to have a ‘warm-up’ excursion phase when placed in novel environments (Thompson et al., 2018). Additionally, the timing of the IRAP inhibitor administration determines which stages of memory most likely to be affected. In this study, the administration of HA08 was made immediately prior to the training phase in the NOR test, focusing the effects on memory acquisition, rather than memory consolidation or recall. Prior literature shows that angiotensin IV can enhance acquisition, consolidation, and recall memory (Gard, 2008), and other IRAP inhibitors, such as HFI-419, have shown improvements when given before memory acquisition (Albiston et al., 2008). Importantly, one major difference between the current behavioural studies and previous cognitive evaluations of IRAP inhibitors is the timing of the tests in regard to the animals’ active or resting phase. Previous studies demonstrating positive effects on cognitive abilities after treatment with IRAP inhibition ligands have performed the behaviour tests during the animals’ resting phase (light phase) (Albiston et al., 2008, Braszko et al., 2006, Braszko et al., 1988, De Bundel et al., 2009, Paris et al., 2013, Pederson et al., 1998, Seyer et al., 2020, Wright et al., 1999), whereas we have evaluated the cognitive performance during the animals active phase (dark phase). The circadian rhythm timing could be a decisive factor for the evaluation of cognitive enhancers, as nocturnal animals often perform cognitive tasks better during their active cycle. Speculatively, small changes in cognitive performance could be more difficult to distinguish during the active cycle, although evaluating behaviour related to memory tasks during the active cycle should be more clinically relevant. Moving forward, studies should further evaluate the impact of performing specific memory tasks during different phases of the light-dark cycle, as well as evaluating the impact of IRAP inhibitors on memory acquisition, consolidation, and recall.

The macrocyclic HA08 might also be limited by metabolic stability, or that the IRAP inhibition induced by this ligand mainly exerts its effects in compromised or damaged systems, suggesting primarily restorative abilities. Another challenge with the macrocyclic substances is the difficulty of them passing the blood brain barrier, adding to the complexity of treatment and *in vivo* effect evaluation. Icv-surgery was performed to enable direct injection of the substance in the brain ventricles which eliminated some of these challenges, although introducing other problematic aspects such as a reduced weight gain in animals undergoing surgery during the first week after surgery, as expected after a surgical procedure. However, no difference in NOR test performance was observed between the surgery and non-surgery animals in our study. It remains a huge challenge to synthesize IRAP inhibitors that are both biologically stable and efficient, and that ideally also crosses the blood brain barrier, which would enable other administration alternatives for behaviour evaluation.

In previous *in vitro* studies, we have shown that the effect of IRAP inhibition differs between hippocampus and cortical tissues (Stam et al., 2024, Stam et al., 2022). Spatial memory tasks are primarily dependent on the hippocampus, whereas recognition memory is suggested to be less reliant on the function of this brain structure (Broadbent et al., 2004). This would suggest that evaluation of the function of IRAP in relation to different cognitive functions are highly dependent on specific areas of the brain. Future investigations of HA08, or other potential IRAP inhibitors, may benefit from including additional behavioral paradigms, with particular attention to the brain areas and networks involved in the targeted cognitive functions. Additionally, disease models or animals with induced cognitive deficiencies are also approaches that should be in focus for future studies to evaluate the *in vivo* effects of IRAP inhibitors, since it may be difficult to measure cognitive improvement in animals with intact memory functions.

## Conclusions

The results of this study demonstrate that an acute single dose of IRAP inhibitor HA08 had no effect on recognition memory acquisition or spatial working memory in male rats with intact cognitive abilities when tested under a reversed light–dark cycle. Although no measurable cognitive effects were demonstrated in this study setup, a decreased locomotor activity was observed for HA08 treated animals in the Y-maze test. Further studies to assess the possible cognitive, and physical, effects of HA08 are warranted, and this inhibitor should still be considered a potent candidate for further evaluation of IRAP as a pharmaceutical target. We suggest that this inhibitor and similar ligands, most likely requires repeated administration to *in vitro-* and *in vivo* systems to induce significant effects. Additionally, using disease models may contribute to fully unravel the prospect and mechanisms of IRAP inhibition as a cognitive enhancing therapy.

## CRediT authorship contribution statement

**Frida Stam:** Conceptualization, Data curation, Formal analysis, Investigation, Methodology, Writing – original draft, Writing – review & editing. **Sara Bjurling:** Data curation, Investigation, Writing – review & editing. **Sofia Zelleroth:** Data curation, Investigation, Methodology, Writing – review & editing. **Sally Badrd'din:** Investigation, Writing – review & editing. **Johan Gising:** Methodology, Writing – review & editing. **Mats Larhed:** Conceptualization, Methodology, Writing – review & editing. **Mathias Hallberg:** Conceptualization, Funding acquisition, Resources, Supervision, Writing – review & editing. **Alfhild Grönbladh:** Conceptualization, Funding acquisition, Investigation, Methodology, Supervision, Writing – review & editing.

## Funding

This project was supported by Kjell and Märta Beijer Foundation, Magnus Bergvall stiftelse, and the Åke Wiberg stiftelse.

## Declaration of Competing Interest

The authors declare that they have no known competing financial interests or personal relationships that could have appeared to influence the work reported in this paper.
